# CRISPR/Cas9-based gene activation and base editing in *Populus*

**DOI:** 10.1093/hr/uhad085

**Published:** 2023-05-05

**Authors:** Tao Yao, Guoliang Yuan, Haiwei Lu, Yang Liu, Jin Zhang, Gerald A Tuskan, Wellington Muchero, Jin-Gui Chen, Xiaohan Yang

**Affiliations:** Biosciences Division, Oak Ridge National Laboratory, Oak Ridge, TN 37831, USA; The Center for Bioenergy Innovation, Oak Ridge National Laboratory, Oak Ridge, TN 37831, USA; Biosciences Division, Oak Ridge National Laboratory, Oak Ridge, TN 37831, USA; The Center for Bioenergy Innovation, Oak Ridge National Laboratory, Oak Ridge, TN 37831, USA; Chemical and Biological Process Development Group, Pacific Northwest National Laboratory, 902 Battelle Boulevard, Richland, WA 99352, USA; Biosciences Division, Oak Ridge National Laboratory, Oak Ridge, TN 37831, USA; Department of Academic Education, Central Community College –Hastings; Hastings; NE 68901, USA; Biosciences Division, Oak Ridge National Laboratory, Oak Ridge, TN 37831, USA; Biosciences Division, Oak Ridge National Laboratory, Oak Ridge, TN 37831, USA; State Key Laboratory of Subtropical Silviculture, College of Forestry and Biotechnology, Zhejiang A&F University; Hangzhou 311300, China; Biosciences Division, Oak Ridge National Laboratory, Oak Ridge, TN 37831, USA; The Center for Bioenergy Innovation, Oak Ridge National Laboratory, Oak Ridge, TN 37831, USA; Biosciences Division, Oak Ridge National Laboratory, Oak Ridge, TN 37831, USA; The Center for Bioenergy Innovation, Oak Ridge National Laboratory, Oak Ridge, TN 37831, USA; Biosciences Division, Oak Ridge National Laboratory, Oak Ridge, TN 37831, USA; The Center for Bioenergy Innovation, Oak Ridge National Laboratory, Oak Ridge, TN 37831, USA; Biosciences Division, Oak Ridge National Laboratory, Oak Ridge, TN 37831, USA; The Center for Bioenergy Innovation, Oak Ridge National Laboratory, Oak Ridge, TN 37831, USA

## Abstract

The genus *Populus* has long been used for environmental, agroforestry and industrial applications worldwide. Today *Populus* is also recognized as a desirable crop for biofuel production and a model tree for physiological and ecological research. As such, various modern biotechnologies, including CRISPR/Cas9-based techniques, have been actively applied to *Populus* for genetic and genomic improvements for traits such as increased growth rate and tailored lignin composition. However, CRISPR/Cas9 has been primarily used as the active Cas9 form to create knockouts in the hybrid poplar clone “717-1B4” (*P. tremula* x *P. alba* clone INRA 717-1B4). Alternative CRISPR/Cas9-based technologies, e.g. those involving modified Cas9 for gene activation and base editing, have not been evaluated in most *Populus* species for their efficacy. Here we employed a deactivated Cas9 (dCas9)-based CRISPR activation (CRISPRa) technique to fine-tune the expression of two target genes, *TPX2* and *LecRLK-G* which play important roles in plant growth and defense response, in hybrid poplar clone “717-1B4” and poplar clone “WV94” (*P. deltoides* “WV94”), respectively. We observed that CRISPRa resulted in 1.2-fold to 7.0-fold increase in target gene expression through transient expression in protoplasts and *Agrobacterium*-mediated stable transformation, demonstrating the effectiveness of dCas9-based CRISPRa system in *Populus*. In addition, we applied Cas9 nickase (nCas9)-based cytosine base editor (CBE) to precisely introduce premature stop codons via C-to-T conversion, with an efficiency of 13%–14%, in the target gene *PLATZ* which encodes a transcription factor involved in plant fungal pathogen response in hybrid poplar clone “717-1B4”. Overall, we showcase the successful application of CRISPR/Cas-based technologies in gene expression regulation and precise gene engineering in two *Populus* species, facilitating the adoption of emerging genome editing tools in woody species.

## Introduction

Commonly known as poplars, cottonwoods, and aspens, the genus *Populus* consists of about 30 tree species naturally occurring in the northern hemisphere. These tree species provide both environmental benefits and industrial supplies. In the United States and many other countries, *Populus* plantations have been established for urban enhancement, water filtration, bioremediation, and agroforestry [1, 2]. They can also be used for carbon sequestration and production of biofuels [3, 4]. Worldwide there are more than 5 million hectares of *Populus* plantations [5].

Because of their diverse usages in landscape, agriculture, bioenergy, and industry, *Populus* species have been the focus of many tree breeding and genetic improvement programs. Modern biotechnologies, including both genomics and genetic engineering (GE), have been considered as versatile tools for accelerating *Populus* domestication. Moreover the release of the *P. trichocarpa* genome sequence in 2006 [6] and the rapid development of next-generation sequencing (NGS) in the past decade facilitate the discovery of the genetic basis of important domestication traits. For example, it was found that in the genome of *P. trichocarpa*, the locus Potri.005G018000, which encodes a G-type lectin receptor-like protein kinase (LecRLK-G), showed strong association with the susceptibility to the invasive fungal pathogen *Sphaerulina musiva* in a genome-wide association study (GWAS) [7]. In addition, photosynthesis or bioenergy production-related genes identified from other species can also be used in tree genetic improvement [8]. One example gene that can benefit *Populus* breeding is *Targeting Protein for Xklp2* (*TPX2*), which belongs to an evolutionally-conserved gene family that regulates microtube dynamics in humans and plants [9]. This gene was also identified as a candidate gene to increase photosynthetic efficiency in C_3_ plants through the analysis of diel gene expression patterns [8].

Since *Populus* species are obligate outcrossing species and are cross-pollinated in natural environment with a long juvenile peroid, GE has great potential for shortening the improvement cycle needed for *Populus*. In *Populus*, GE often relies on *Agrobacterium*-mediated DNA delivery and *in vitro* regeneration of plantlets from cells in which exogenous DNA is integrated into the genome. Among the several different *Populus* species or clones that can be manipulated by GE, the hybrid poplar clone “717-1B4” (*P. tremula x P. alba* INRA 717-1B4) has been most widely used due to the availability of well-established systems for *Agrobacterium*-mediated transformation [10, 11]. Alternatively, the poplar clone “WV94” (*P. deltoides* “WV94”) is favored in genetic improvement programs and scientific research due to the availability of reference genome sequence, high stress tolerance, rapid growth rate, and high biomass yield [12–16].

The discovery of the mechanisms of clustered regularly interspaced short palindromic repeats (CRISPR) and CRISPR-associated protein (Cas) systems in bacterial immune systems has revolutionized the field of eukaryotic genome engineering [17–19]. CRISPR-induced genome editing involves the generation of a Cas9-induced double-strand break that is repaired by non-homologous end joining (NHEJ) mechanisms or by homology directed repair (HDR) if a donor template is provided [19]. The most widely used programmable RNA-guided DNA endonuclease for genome editing is the type II SpCas9 system derived from *Streptococcus pyogenes*, which has been applied for targeted mutagenesis in different plant species [18, 20–22]. CRISPR/Cas9-mediated mutagenesis has also been widely applied in poplars, mainly in the hybrid poplar clone “717-1B4” due to its transformation capacity [23, 24]. In addition, knockout mutants can be generated effectively in poplar in the primary transformants [25].

Motivated by the need of precision genome editing, gene regulation and other types of genome engineering, alternative CRISPR/Cas9-based genome engineering tools, such as CRISPR-based activators, CRISPR-based inhibitors, base editors, and prime editors, have been developed using dCas9 (D10A and H840A) or nCas9 (D10A or H840A) [26–29]. Different dCas9-based activators have been generated and applied for activating gene expression in different plant species [27, 30]. In particular, the most recently designed system named CRISPR-Act3.0 has been reported to be highly efficient in activating genes in *Arabidopsis*, rice, and tomato [31, 32]. The base editor A3A-PBE, consisting of a nCas9 and the human APOBEC3A, has been shown to be able to convert cytidine to thymidine efficiently in wheat, rice, and potato with a 17-nucleotide editing window [26].

The development of alternative CRISPR-related tools, such as CRISRPa and base editing, has demonstrated great potential for improving plant fitness. Using CRISPR/dCas9-based gene activation tool kit, the *ZmBBM2* gene was activated precisely in the egg cells that can be used for parthenogenesis induction in maize [33]. Activation of the *AREB1* gene by CRISPR/dCas9 fused with a histone acetyltransferase was shown to enhance plant drought tolerance in *Arabidopsis* [34]. The CRISPR/dCas9-TV transcriptional activator up-regulated the expression of *CBF4* and consequently increased cold tolerance in grape [35]. Base editing can generate single-point mutation at designated amino acid to change protein functions. *Arabidopsis eIF4E1* gene was modified by CRISPR/nCas9-cytidine deaminase, resulting in enhanced resistance to potyviruses [36]. In summary, these new CRISPR tools are capable of precisely adjusting gene function that can be used to improve plant performance and fitness. Yet the application of CRISPRa and base editing in *Populus* remains very limited. In this study, we developed a screening system based on GUS and LUC reporters, which enables the efficient identification of highly effective single-guide RNAs (sgRNAs) in protoplasts. Additionally, we investigated the potential of CRISPR/Cas9-based activators and base editors in two *Populus* species. Our findings expanded the application of CRISPRa and base editing techniques in woody species.

## Results

### Development of a high-throughput reporter gene-based CRISPRa evaluation system in protoplasts

**Figure 1:**
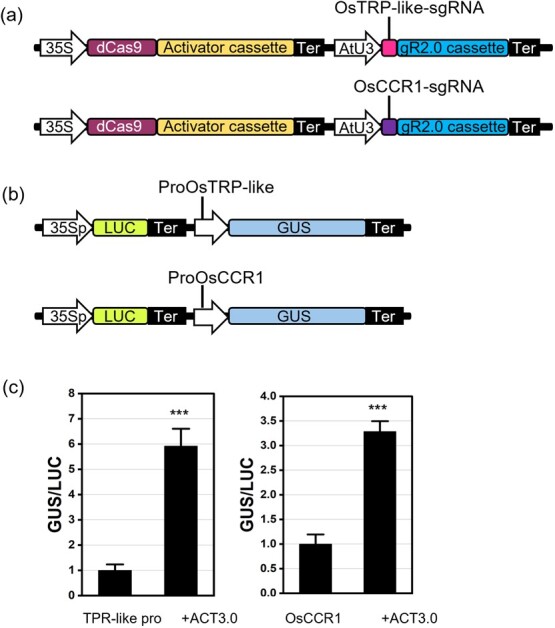
GUS/LUC dual reporter system for detecting CRISPRa efficacy. (**a**) CRISPRa construct design for *OsTRP-like* and *OsCCR1* genes. (**b**) Reporter construct design for *OsTRP-like* and *OsCCR1* promoters. (**c**) CRISPRa efficiency measurement using two reporter genes, *GUS* and *LUC*, in *Arabidopsis* protoplasts. Bar charts represent mean ± sd (n = 3 three independent protoplast pools), and asterisks represent significant difference between groups determined by Student’s *t*-test: ^***^, *P* ≤ 0.001.

CRISPRa efficiency can be variable according to different activators employed and sgRNA selection [27, 30, 37]. The effectiveness of CRISPRa is assessed by the induction of target gene expression. Here, we developed an alternative reporter-gene-based CRISPRa efficiency evaluation system. There are two constructs in this screening system, the activation construct and the reporter construct. The activation construct consists of dCas9-based activator cassette and gRNA cassette, which is the same vector used for generating CRISPRa transgenics (Fig. 1a). The reporter construct consists of *promoter::GUS* reporter cassette and *35S::LUC* reference cassette (Fig. 1b). By co-transfecting of activation construct and reporter construct in protoplasts, the activation efficiency can be evaluated by GUS enzyme activity normalized by the luciferase activity.

To assess and improve this evaluation system, we adopted the CRISPR-Act3.0 system with sgRNAs for activating *OsTRP-like* gene or *OsCCR1* gene in rice protoplasts [27]. The effectiveness of this system on gene activation can also be verified using the fluorescent marker in *Arabidopsis* protoplasts. Consistent with previous report [27], mCherry reporter system in *Arabidopsis* protoplasts showed the efficacy of this activation system (Figure S1). Meanwhile, we cloned the promoters of *OsTRP-like* or *OsCCR1* genes to generate reporter constructs (Fig. 1a and b). By co-transfection of *OsTRP-lik*e activation vector and its reporter vector in *Arabidopsis* protoplasts, we measured the activities of GUS and LUC enzymes and calculated the ratio. As shown in Fig. 1c, with the OsTRP-like activation vector, the ratio of GUS/LUC is six-fold higher than those without the activation vector. Similar results were also acquired for *OsCCR1* gene (Fig. 1c). Therefore, we successfully built a quantitative CRISPRa efficiency evaluation system that is reflected by the ratio of GUS and LUC enzyme activities. Since the measurements were taken in the 96-well micro-plate reader, the tested evaluation system can be performed in a high-throughput manner to facilitate the screen of sgRNAs.

**Figure 2:**
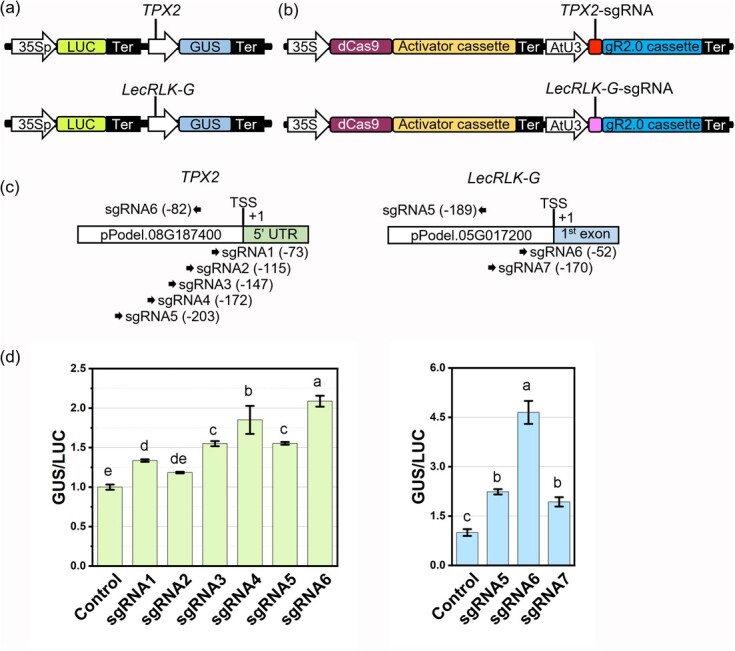
SgRNA selection for high-efficient CRISPRa in poplar. (**a**) Reporter construct design for TPX2 and LecRLK-G promoters. (**b**) CRISPRa construct design for *TPX2* and *LecRLK-G* genes. (**c**) SgRNA distributions on the promoters of *TPX2* and *LecRLK-G*. (**d**) CRISPRa efficiency measurement for individual sgRNAs in poplar *“*WV94” protoplasts. For the control samples, the effector plasmids lacking sgRNA were used. Bar charts represent mean ± sd (n = 3 three independent protoplast pools), and different letters represent significant difference between groups (*P* ≤ 0.05) determined by one-way ANOVA followed by Tukey’s test.

### Screening sgRNAs for gene activation in *Populus* protoplasts

To apply the CRISPR-Act3.0 system in activating *Populus* endogenous genes, we selected two target genes, *TPX2* and *LecRLK-G*, as case studies. These genes were reported to be involved in photosynthesis and microtubule dynamics, and plant immunity, respectively [7–9]. To get high-efficiency gene activation, various sgRNAs were screened in *Populus* protoplasts using the GUS/LUC dual reporter system we developed. First, we cloned 500-bp of promoter regions of *TPX2* and *LecRLK-G* genes from poplar clone “WV94” (*Populus deltoides* “WV94”) and inserted them into reporter constructs (Fig. 2a and b). Second, we designed the sgRNAs using the CHOPCHOP online tool. Based on predicted on-target efficiency, number of off-targets, GC content and self-complementarity, six and three sgRNAs, located 0- to -200-bp regions from the TSS, were selected for *TPX2* and *LecRLK-G* genes, respectively (Fig. 2c). These sgRNAs were individually cloned into the CRISPR-Act3.0 vector and assessed for their activation abilities.

By co-transfecting the reporter constructs and the activation construct containing each sgRNA, the activation efficiency of each sgRNA was assessed by GUS enzyme activities. The reference expression cassette *35S::LUC* was incorporated into the reporter constructs as the internal controls, and the activation efficiency was quantified by the ratio of GUS and LUC expression levels. Among six sgRNAs of *TPX2*, the GUS activities were increased by 1.2- to 2.1-fold, and sgRNA6 outperformed the other sgRNAs (Fig. 2d). Likewise, three sgRNAs of *LecRLK-G* also showed varied activation efficiency from 1.9- to 4.7-fold, and sgRNA6 containing activation construct showed the highest activation efficiency (Fig. 2d). Therefore, both the sgRNA6 of *TPX2* and the sgRNA6 of *LecRLK-G* were selected for further stable transformation studies.

### Establishment of stable CRISPRa transgenic plants in poplar

**Figure 3:**
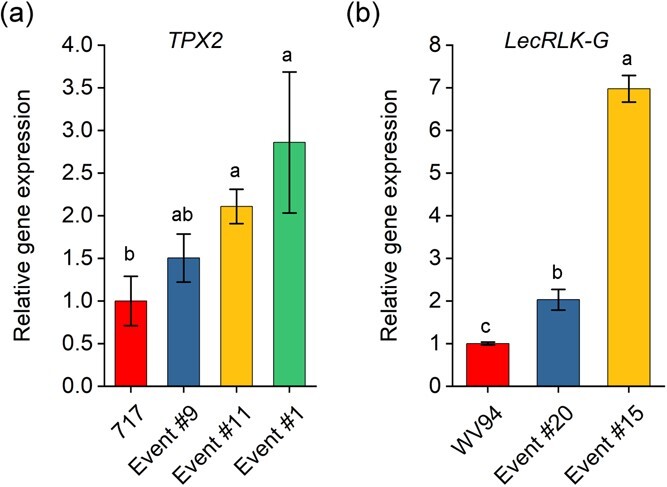
Gene activation efficiency analyses in stable transgenic *Populus*. (**a**) Expression of *TPX2* in three independent transgenic events, Event #9, Event #11 and Event #1 in hybrid poplar clone “717-1B4” plants. (**b**) Expression of *LecRLK-G* in two independent transgenic events, Event #20 and Event #15 in poplar clone *“*WV94” plants. Bar charts represent mean ± se (n = 3 three independent plants), and different letters represent significant difference between groups (*P* ≤ 0.05) determined by one-way ANOVA followed by Tukey’s test.

To further examine the CRISPRa effectiveness in stable transgenic poplars, we chose two *Populus* genotypes for generating CRISPRa lines. Since the selected promoter regions of *TPX2* in clone “WV94” and clone “717-1B4” (*P. tremula* x *P. alba* clone INRA 717-1B4) for sgRNA design are conserved, the sgRNA6 is expected to work in clone “717-1B4” as well (Fig. S2). *Agrobacterium*-mediated leaf-disc transformation was used to transform *CRISPRa-TPX2* vectors into hybrid poplar clone “717-1B4” plants. Three transgenic events were retrieved from the transformation and subject to CRISPRa efficiency evaluation (Fig. S3). Using *TPX2* gene specific primers, the transcript abundance of *TPX2* was analyzed by RT-qPCR assays. Compared to wild-type plants, three transgenic events showed elevated gene expression levels from 1.5-fold to 2.9-fold (Fig. 3a). In addition, we transformed CRISPRa construct with sgRNA6 of *PtLecRLK-G* gene into clone “WV94”. Two independent events were retrieved from the transformation and the gene activation efficiency was examined (Fig. S4). RT-qPCR results showed that the expression of *LecRLK-G* gene was increased by 2-fold and 7-fold in Event #20 and Event #15 as compared to the wild-type plants, respectively (Fig. 3b). These results demonstrate that the CRISPR-Act3.0 system is capable to activate endogenous *TPX2* or *LecRLK-G* genes in stable transgenic poplars.

### Installation of premature termination codons using base editing in poplar

**Figure 4:**
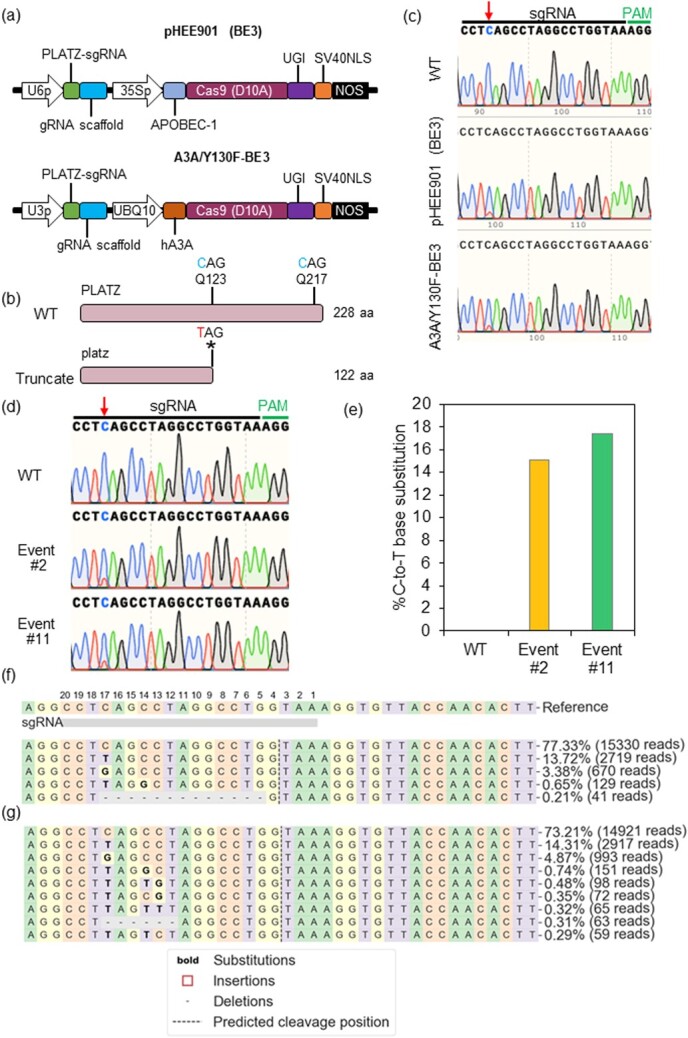
Application of cytosine base editors in poplar. (**a**) Illustration of cytosine base editors targeting *PLATZ* gene. (**b**) Targeting site design for installation of a stop codon within *PLATZ* gene. (**c**) Sequencing chromatograms of “deamination window” of two poplar “717-1B4” protoplast pools treated with different cytosine base editors as described in panel a. The red arrow indicates the heterozygous peaks. (**d**) Sequencing chromatograms of “deamination window” of two transgenic poplar “717-1B4” events (#2 and #11) harboring C to T mutations. Red arrow indicates the heterozygous peaks. (**e**) NGS analysis of the expected base editing efficiencies in two transgenic events (#2 and #11). (**f**) Sequence profile of transgenic Event #2. (**g**) Sequence profile of transgenic Event #11. The nucleotide upstream of PAM site was labelled with positions 1 to 20.

In previous research, we identified gene *PLATZ* (plant AT-rich protein and zinc-binding protein) which is associated with numerous disease-related genes based on the analysis of the resistant genotype BESC-22 and the susceptible genotype BESC-801 to *Sphaerulina musiva* in *P. trichocarpa* [7, 38]. To study the genetic function of this gene, we aim to create a truncated PLATZ protein by installing a premature termination codon (PTC) via base editing in poplar clone “717-1B4”. With the development of CRISPR technologies, multiple generations of base editors have been reported and applied in plant research. Based on the relatively high efficiency, we selected two base editors, pHEE901(BE3) and A3A/Y130F-BE3 (Fig. 4a) [32, 39]. Using BE-Designer-CRISPR RGEN Tools, we identified two potential sites (Q123 and Q217) and designed the corresponding sgRNAs for installing PTCs. Q123 was further selected as the target site because it is closer to start codon and therefore its mutation more likely to produce truncated PLATZ proteins (Fig. 4b). Next, we conducted protoplast transformation in hybrid poplar clone “717-1B4” to test the efficacy of two base editors and the sgRNA. The transfection efficiency for *Populus* protoplasts is about 60–80%. In comparison with the wild type, the expected point mutation (C to T) was detected both in the protoplasts treated with pHEE901(BE3) and A3A/Y130F-BE3 based on Sanger sequencing (Fig. 4c). More specifically, double peaks indicating C and T were observed in the two samples containing base editors though the peak of T is lower than that of C, but not in the wild type (Fig. 4c).

Since the editing efficiency of two base editors did not show a significant difference in protoplast transformation, we only used pHEE901(BE3) for the stable transformation in poplar. Around 10% of transformation rate was acquired after regeneration (Fig. S5). Among them, 25 transgenic events were generated and confirmed by PCR genotyping. Two events (#2 and #11) showed double peaks detected by Sanger sequencing, accounting for 8% editing efficiency, whereas the non-transgenic WT plants recovered from *Agrobacterium*-mediated transformation did not show any editing events (Fig. 4d). Next-generation sequencing (NGS) was conducted to further confirm the editing events resulting from stable transformation. 15.1% or 17.4% C-to-T base substitutions in the desired site were detected in two transgenic events with no base pair change in wild-type sample (Fig. 4e). Most of the C-to-T substitutions happened at C17 on the protospacer. C14 and C13 have also been edited at a lower efficiency. The C-to-G substitution has also been induced within the editing window at a rate of around 1%. The rate of indel byproduct caused by the pHEE901(BE3) is lower than 0.5% (Fig. 4f,g). Therefore, both base editors, pHEE901(BE3) and A3A/Y130F-BE3, were capable of inducing C-to-T mutation in the *Populus* protoplast system, and pHEE901(BE3) also showed expected base editing in stable transgenic poplars.

## Discussion

To evaluate the efficacity of CRISPR-based technologies in genetic improvement of *Populus*, we tested two technologies – CRISPRa and base editing – in two *Populus* clones “717-1B4” (*Populus tremula* x *P. alba* clone INRA 717-1B4) and “WV94” (*P. deltoides* “WV94”). For gene activation, we adopted the dCas9-based CRISPR-Act3.0 system reported by Pan et al. (2021) to increase the expression of *Populus* endogenous genes. We observed an average activation rate of two-fold for the endogenous gene *TPX2* in clone “717-1B4”, and an average activation rate of four-and-half-fold for the endogenous gene *LecRLK-G* (Podel.05G017200) in clone “WV94” (Fig. 3). The observed activation rates fall within the range previously reported in transgenic rice seedlings, where CRISPR-Act3.0 conferred a 2.0- to 20-fold activation in six different target genes [27]. Much higher rates – up to 140-fold increase – were obtained with the same system in rice, using protoplast cells and transgenic callus [27]. In conclusion, we have demonstrated the effectiveness of dCas9-based CRISPRa system in different *Populus* genotypes for the first time. Future studies will be needed to compare the gene activation efficiency of this CRISPR activation system on different target genes, including *TPX2*, *LecRLK-G*, and other genes among different *Populus* species. Furthermore, with the establishment of this system in hand, screening for additional high-efficient sgRNAs or using multiple sgRNAs to target the same promoter could be a promising strategy to activate endogenous gene expression.

In screening sgRNAs for their activation efficiency, we adopted a GUS/LUC reporter system which has been commonly used to study the effects of transcription factors [40]. To tailor this GUS/LUC system for sgRNA screening, we cloned the promoter of the gene of interest (*i.e.* the gene to be activated by CRISPRa) into the upstream region of the *GUS* gene, so that *GUS* expression was controlled by the promoter and affected by the efficiency of sgRNAs. Meanwhile, a constitutively expressed LUC was used as a reference. In protoplast-based analysis, this GUS/LUC system estimated an activation range of two-fold for the sgRNA6 of *TPX2* and an activation rate of four-and-half-fold for the sgRNA6 of *LecRLK-G* (Fig. 2). The estimated rates were comparable with what we observed in stably transformed *Populus* events using RT-qPCR, suggesting high reliability of this GUS/LUC protoplast system in quantitive prediction of sgRNA efficiency in stable transgenics (Fig. 3). Compared to detecting endogenous gene activation by RT-qPCR or RNA-seq, the GUS/LUC reporter system can be performed in a high-throughput and cost-effective manner. Our results also show the potential of adopting similar dual reporter systems, the firefly LUC/renilla LUC, which was recently used for quantitative analysis of promoter activities in plants, for estimating the activation effects of sgRNAs [41, 42].

The observed gene activation in this study indicates that this level of gene activation may confer phenotypic changes. For many genes, a modest change in expression level can lead to phenotypic modifications in *Populus* and other plant species [43, 44]. For example, in the hybrid poplar clone “NL 895” (*P. deltoids* x *Populus euramericana* clone “NL 895”), a four-fold activation in the expression of *PdeGATA3*, which encodes a GATA transcription factor, was able to produce dwarfism traits, including reduced leaf size, internode length, petiole length, and plant height, under tissue culture and greenhouse conditions [44]. Furthermore, this modest change in the expression of *PdeGATA3* affected the expression of other genes, including the SHOOT MERISTEMLESS encoding gene *PdeSTM* and the GA20oxidase encoding gene *PdeGA20ox* [44]. Similarly, in *Eucalyptus,* it was found that an average of four-fold higher expression of the *Arabidopsis FLOWERING LOCUS T* (*AtFT*) gene was detected in the flowering group compared with the non-flowering group, when analyzing the correlation between ectopic expression of *AtFT* and the flowering trait [43]. Considering that we observed an activation effect as high as seven-fold in one of the *Populus* transgenic events (Fig. 3b), we could expect phenotypic changes in follow-up studies.

We also demonstrated that both APOBEC1-BE3 and A3A/Y130F-BE3 can introduce C-to-T base changes in clone “717-1B4” and that pHEE901(BE3) could edit a window from C17 to C13 on the protospacer at the designated target site, which is smaller compared with the editing window of A3A/Y130F-BE3 in poplar [32]. Only a small number of C-to-G substitutions and indels byproducts were induced by the pHEE901(BE3), indicating the reliability of this base editor (Fig. 5). Two chimeric base edited events were obtained from twenty-five transgenic lines in this study, indicating a low editing efficiency of pHEE901(BE3) at this target site. It has been shown that A3A/Y130F-BE3 and PmCDA1-BE3 can result in a wide range base editing frequency from 0% to 100% across different target sites [32]. To further evaluate the efficiency of pHEE901(BE3) in poplar, more target sites may need to be investigated. Since only chimeric mutants were generated in this study, a second round of shoot regeneration can be used to obtain homozygous mutants from these chimeric mutants [18, 45]. The present study has provided a foundation for testing additional higher efficiency base editors in *Populus* to generate homozygous base edited mutants. The present study has established the technical platform for generating CRISPR-based activation and base editing in a perennial woody species. In future studies, phenotypic characterization of these activation and base edited lines are expected to provide further information about the application potential of this genomic editing techniques in woody plants including poplar and other horticultural plants.

In summary, we tested the application of dCas9-based CRISPRa system in two *Populus* species and demonstrated its effectiveness. We also employed two nCas9-based base editors for precise C-to-T base editing studies. These results not only shed lights on the broad adoption of novel CRISPR/Cas9-based genome editing technology in poplar, but also provide technical concepts for tree breeding program through GE approaches.

## Materials and methods

### Plant materials

*Arabidopsis* wild-type Col-0 plants were grown in soil within the growth chamber with 12 light/ 12 h dark period with light intensity of 100 μmol m ^−2^ s ^−1^ at 21°C. The *in vitro* grown hybrid poplar clone “717-1B4” (*P. tremula* x *P. alba* clone INRA 717-1B4) and poplar clone “WV94” (*P. deltoides* “WV94”) plants were maintained in MS medium in a growth room with 16 h light/8 h dark period with light intensity of 100 μmol m ^−2^ s ^−1^ at 25°C. The plants were sub-cultured into a fresh medium monthly.

### SgRNA design and vector construction

SgRNA design for CRISPRa and base editing were performed with CHOPCHOP and BE-Designer-CRISPR RGEN online tools, respectively [46, 47]. The vectors *ProOsTRP-like::mCherry*, *ProOsCCR1::mCherry*, *CRISPR-Act3.0-ProOsTRP-like* and *CRISPR-Act3.0-ProOsCCR1* have been described previously [27]. The GUS/LUC dual reporter vector was created by inserting a PCR amplified LUC expression cassette into a GUS vector using NEBuilder® HiFi DNA Assembly [40]. The vectors *ProOsTRP-like::GUS/LUC* and *ProOsCCR1::GUS/LUC* were created by inserting PCR amplified *ProOsTRP-like* and *ProOsCCR1* fragments into 5’-*GUS* gene of GUS/LUC vector using NEBuilder® HiFi DNA Assembly. Same procedures were used to create vectors *ProTPX2::GUS/LUC* and *ProLecRLK-G::GUS/LUC*. CRISPR-Act3.0-TPX2 (sgRNA1–6) and CRISPR-Act3.0-LecRLK-G (sgRNA5–7) were constructed by inserting PCR amplified three fragments, AtU3 promoter, corresponding sgRNA, and rbcS-E9t terminator, into the vector pLR4061 [27]. For base editing vectors, we cloned the full fragment of *pHEE901(BE3)* [39] into a *pGFPGUSplus_KAN* vector [48] to create a kanamycin resistant vector *BE_KAN*. Then a gBlock of *PLATZ-sgRNA* was synthesized and inserted into BE_KAN vector using NEBuilder® HiFi DNA Assembly to create vector *BE_KAN_PLATZ*. Similarly, we created a kanamycin resistant vector *pK_HA3A_CBE* by inserting two fragments from pLR2371 [32] into vector *pKSE401(AtU3)* [49]. Then *PLATZ-sgRNA* was inserted to vector *pK_HA3A_CBE* via Golden Gate Assembly to create *pK_HA3A_CBE_PLATZ*.

### Protoplast transformation

*Arabidopsis* and *Populus* protoplasts were isolated as previous reports [50, 51]. In brief, full expanded leaves from one-month-old *Arabidopsis*, poplar clone “WV94” and hybrid poplar clone “717-1B4” plants were sliced into strips and immersed into enzyme solution (0.4 M mannitol, 20 mM KCl, 20 mM MES, 10 mM CaCl_2_, 5 mM β-mercaptoethanol, 0.1% BSA, 0.8% macerozyme R10, and 3% cellulase R10). After 3–5 hours, the protoplasts were filtered by a 75 mm nylon mesh and washed with W5 solution (154 mM NaCl, 125 mM CaCl_2_, 5 mM KCl, and 2 mM MES). After centrifuging at 1000 rpm for 3 min at 4°C, the protoplasts were resuspended with MMg solution (0.8 M mannitol, 1 M MgCl_2_ and 0.2 M MES) at the concentration of 200, 000–400, 000 cells per mL.

Ten μg of CRISPRa and reporter plasmids, and 100 ng reference plasmids were co-transfected into 100 μL of *Arabidopsis* or *Populus* protoplasts using PEG/Ca^2+^ solution (100 mM CaCl_2_, 0.2 M mannitol, 40% PEG4000). For the control samples, the CRISPRa plasmids lacking the sgRNAs were used. After incubating in W5 solution for 16–20 hours, the protoplasts were harvested for GUS/LUC enzyme activity analysis. Ten μg of the base editor constructs were transfected in to 100 ml of *Populus* protoplasts using PEG/Ca^2+^ solution (100 mM CaCl_2_, 0.2 M mannitol, 40% PEG4000). After 15 min, the transfected protoplasts were washed and incubated in W5 solution for 48 h before DNA extraction.

### GUS/LUC enzyme activity measurements

GUS enzyme activities in protoplasts were measured by a Fluoroskan microplate reader using the substrate MUGlcU (4-Methylumbelliferyl β-D-Glucuronide) (M1490, ThermoFisher). *35S::LUC* plasmids were use a reference, and the enzyme activity of LUC was measured by the Luciferase Assay Systems (E1500, Promega) according to the manufacturer’s instructions.

### *Agrobacterium*-mediated plant stable transformation

Hybrid poplar clone “717-1B4” plants were transformed using *Agrobacterium*-mediated leaf-disc transformation [11]. The transformants were selected in calli induction medium (MS medium containing 10 μM NAA and 5 μM 2ip), shoot selection medium (MS medium containing 0.2 μM TDZ), shoot elongation medium (MS medium containing 0.1 μM BAP), and rooting medium (1/2MS medium containing 0.5 μM IBA). Poplar clone “WV94” was transformed using a modified *Agrobacterium*-based method and transgenic events were generated through shoot induction, shoot elongation and root induction on Broadleaf Tree Basal Medium (PhytoTech Labs) supplied with hormones and antibiotics [13].

Timentin (200 mg/L) and cefotaxime (300 mg/L) were included to inhibit the *Agrobacterium* growth. Kanamycin (100 mg/L) were used to select positive transformants. Rooted transgenic events were genotyped using PCR to verify the presence of CRISPRa or base editing constructs.

### RNA extraction and quantitative reverse transcription PCR (RT-qPCR)

Plant total RNAs were extracted from leaves of one-month-old soil-grown poplars using a Sigma plant total RNA kit according to the manufacturer’s instructions. Two micrograms of RNAs were reverse transcribed in to cDNAs using a SuperScript III kit and oligo (dT) 18 as primers (Invitrogen). RT-qPCR was carried out using Maxima SYBR Green/ROX qPCR Master Mix (ThermoFisher Scientific) and the reference and gene-specific primers are listed in Supplementary Table S1.

### DNA sequencing

The plasmids and PCR products were Sanger sequenced using SimpleSeq Kit Premixed (Eurofins Genomics) [48]. For genotyping the based editing cells or plants, target regions were amplified by PCR and sequenced by Sanger sequencing and next generation sequencing. For NGS sample preparation, DNA was extracted from leaf of transgenic plants using modified SDS method. Gene-specific primers were used for amplifying *PLATZ* target. Q5 high-fidelity polymerase (New England Biolabs) was used for amplifying the target DNA region with the following PCR cycling conditions: 98°C for 30 s, 35 cycles of 98°C for 10 s, 65°C for 30 s, and 72°C for 30 s, with the final elongation step at 72°C for 2 min. The PCR products were separated in the 2% agarose gel, and target bands were excised and extracted. Next Generation Sequencing (NGS) was used to sequence amplicons via GENEWIZ Amplicon-EZ services. Mutations were assessed for each sample using CRISPResso2 [52]. Primers are listed in the Supplementary Table S1.

## Acknowledgements

We thank Dr. Yiping Qi from University of Maryland for providing the CRIPSR-Act3.0 and A3A/Y130F-BE3 related vectors. We thank Miranda Clark, David McLennan, and Jamie McBrien for growing and maintaining plants in ORNL greenhouses. This work was supported by the Center for Bioenergy Innovation, a U.S. Department of Energy (DOE) Bioenergy Research Center supported by the Biological and Environmental Research program and the DOE Genomic Science Program, as part of the Secure Ecosystem Engineering and Design Scientific (SEED) Focus Area and the Plant-Microbe Interfaces (PMI) Scientific Focus Area. Oak Ridge National Laboratory is managed by UT-Battelle, LLC for the U.S. Department of Energy under Contract Number DE-AC05-00OR22725.

## Author contributions

G.A.T., J-G.C. and X.Y. conceived the research. T.Y., G.Y., H.L. executed and analyzed the experiments. T.Y., G.Y., H.L., and Y.L. drafted the manuscript. J.Z., W.M., G.A.T., J-G.C., and X.Y. revised the manuscript. W.M., J-G.C. and X.Y. supervised the research. All authors have read and agreed to the published version of the manuscript.

## Data availability

The authors confirm that all data from this study are available and can be found in this article and in supplementary information. The plasmids will be available at Addgene.

## Conflict of interest

The authors declare no competing interests.

## Supplementary data

Supplementary data is available at *Horticulture Research* online.

## Supplementary Material

Web_Material_uhad085

## Notes

Notice: This manuscript has been authored by UT-Battelle, LLC under Contract No. DE-AC05-00OR22725 with the U.S. Department of Energy. The United States Government retains and the publisher, by accepting the article for publication, acknowledges that the United States Government retains a non-exclusive, paid-up, irrevocable, world-wide license to publish or reproduce the published form of this manuscript, or allow others to do so, for United States Government purposes. The Department of Energy will provide public access to these results of federally sponsored research in accordance with the DOE Public Access Plan (http://energy.gov/downloads/doe-public-access-plan).
