# Supplementary material for: CRISPR/Cas9-based gene activation and base editing in Populus
Source: Hortic Res. 2023 May 5;10(6):uhad085. doi: 10.1093/hr/uhad085 (PMC10266945; doi:10.1093/hr/uhad085)
Supplement: Web_Material_uhad085 [file web_material_uhad085.zip › Supplementary figures_Yao et al_0322.docx]

**
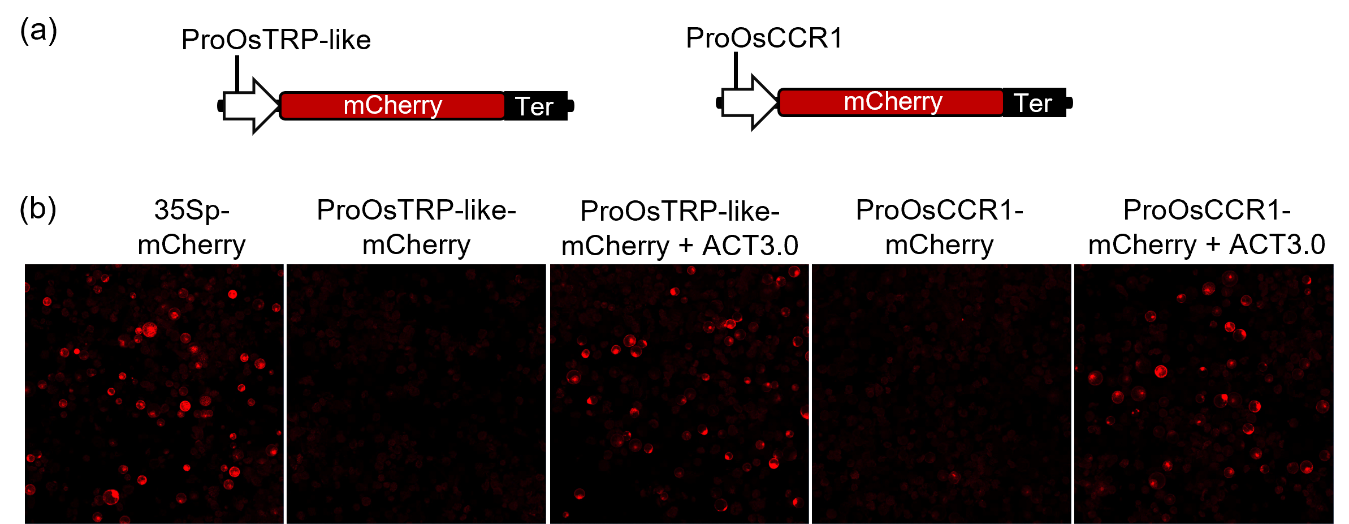
**

**Fig. S1 mCherry reporter system for detecting CRISPRa efficacy.** (**a**) Illustration of mCherry reporter driven by target promoter *ProOsTRP-like* and *ProOsCCR1*, respectively. (**b**) Detection of mCherry signals without and with the CRISPR–Act3.0 activation system in *Arabidopsis* protoplast cells. The mCherry signals were detected using a fluorescence microscope 24 hours after *Arabidopsis* protoplast transformation.


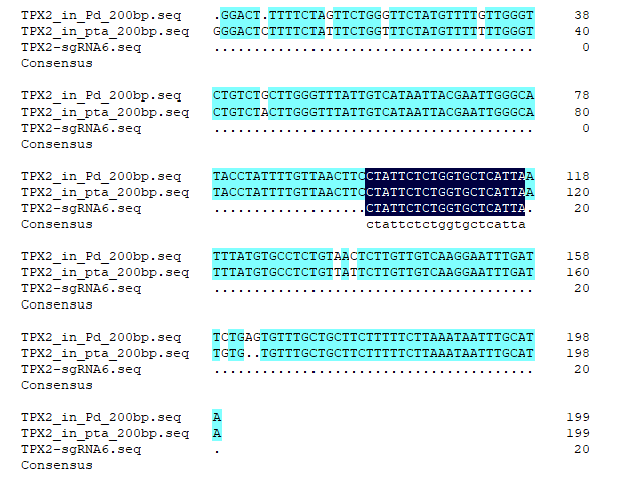


**Fig. S2 Locations of sgRNA6 in the promoter regions of TPX2 in poplar clones of ‘WV94’ and ‘717-1B4’.** The 200-nt promoter sequences of TPX2 before TSSs were aligned, and the sgRNA6 is highlighted.


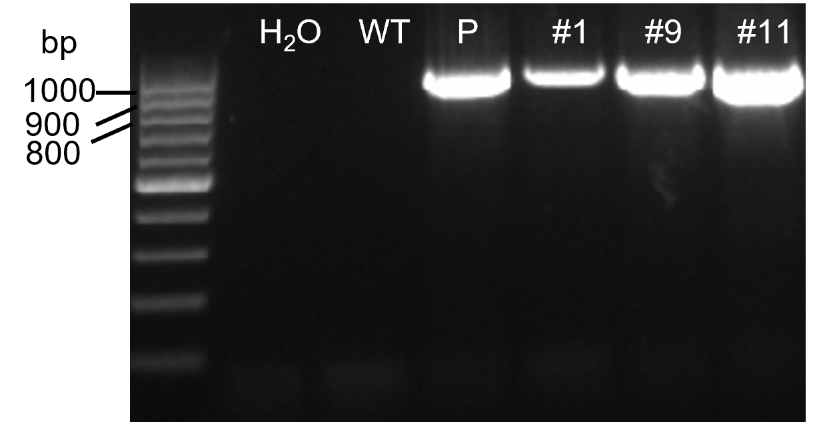


**Fig. S3 Genotyping of *CRISPRa-TPX2* transgenic plants in poplar clone ‘717-1B4’.** The bands represent 877-bp PCR products using *zCAS9* gene-specific primers. WT, wild type; P, positive control.


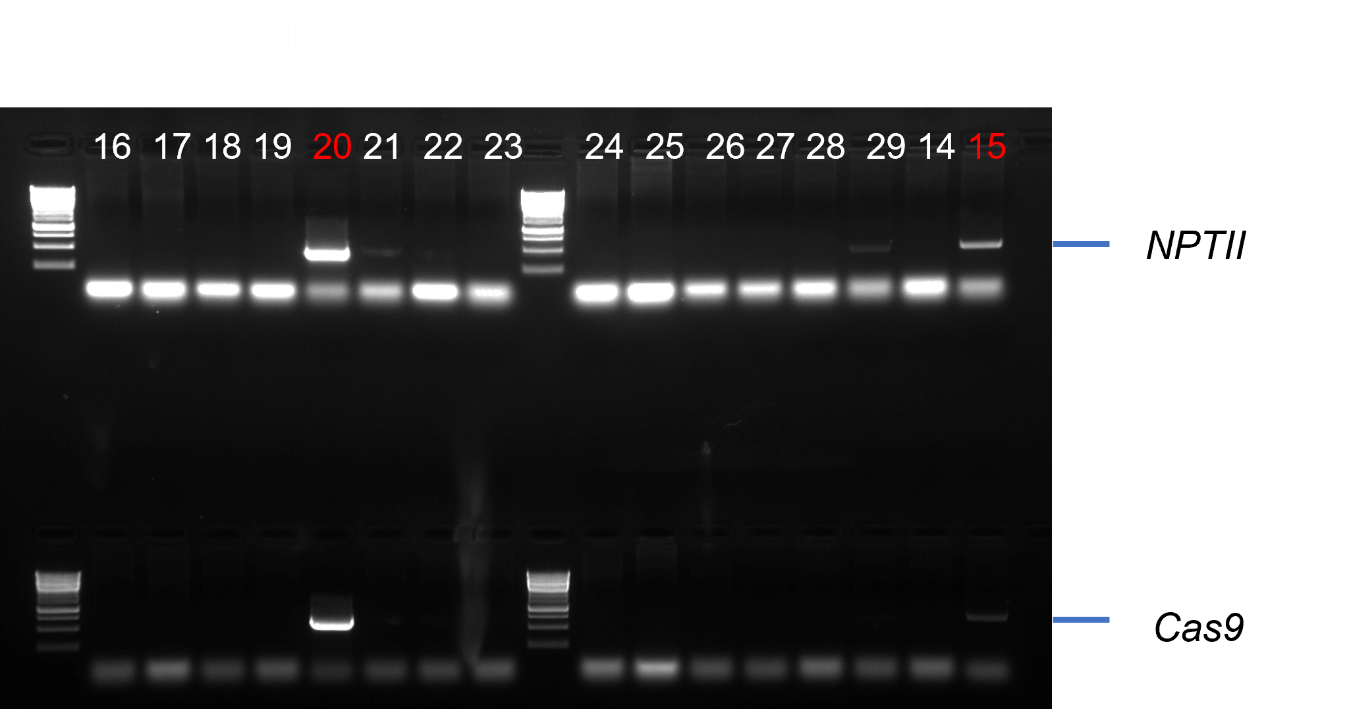


**Fig. S4 Genotyping of *CRISPRa-LecRLK-G* transgenic plants in poplar clone ‘717-1B4’.** The bands in the upper panel represent the PCR products using *NPTII* gene-specific primers. The bands in the lower panel represent the PCR products using *zCas9* gene-specific primers.


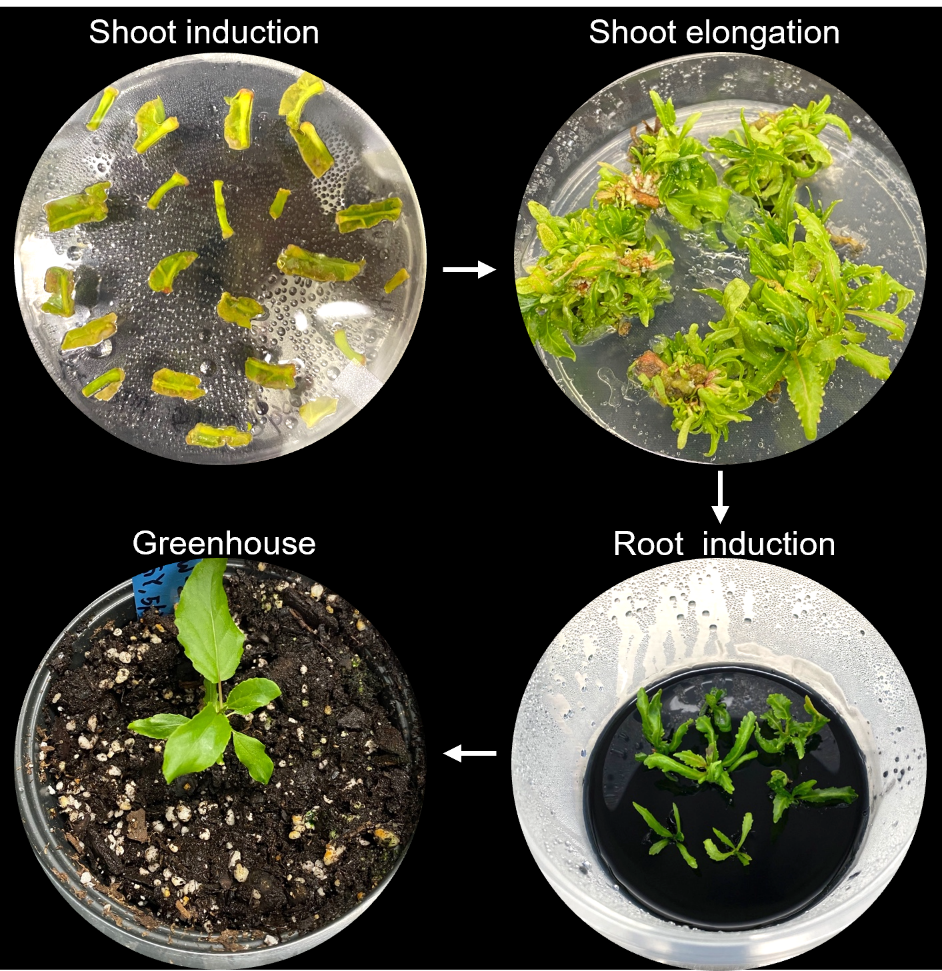


**Fig. S5 Illustration of** **generating transgenic poplar plants.** Leaf and petiole explants were co-cultured with Agrobacteria carrying the base-editing constructs. After shoot induction, shoot elongation, and root induction steps, the transformants were genotyped by PCR and transplant into soil.
